# Understanding the mediation effects of cigarettes per day on time to first cigarette and carcinogen biomarkers: National Health and Nutrition Examination Survey 2015–2016

**DOI:** 10.18332/tid/187766

**Published:** 2024-06-10

**Authors:** Wenxue Lin

**Affiliations:** 1 Department of Epidemiology and Biostatistics, College of Public Health, Temple University, Philadelphia, United States

**Keywords:** volatile organic compounds, time to first cigarette, cigarettes smoked per day, mediation, NHANES

## Abstract

**INTRODUCTION:**

Previous research indicates that cigarette smokers with a time to first cigarette (TTFC) of less than 30 minutes after waking up had significantly higher levels of carcinogen biomarkers compared to those with a TTFC of more than 30 minutes. The mediation (potential mediator: cigarettes smoked per day) between TTFC and carcinogen biomarkers, remains unclear and has yet to be established.

**METHODS:**

Multivariable linear regression models were used to estimate adjusted geometric means (GMs) and ratios of GMs for urine biomarkers of VOCs by smokers’ TTFC status (≤30 vs >30 min). Further, data from the NHANES 2015–2016 special sample were analyzed to assess the mediation between TTFC (exposure) and carcinogen biomarkers, including urine metabolites of polycyclic aromatic hydrocarbons (PAHs), volatile organic compounds (VOCs), and cadmium.

**RESULTS:**

Cigarette smokers with a short TTFC (≤30 min) presented significantly higher concentrations in 8 out of 17 urine metabolites of VOCs examined compared to smokers with TTFC >30 min. The association between exposure and carcinogen biomarkers was not mediated by CPD.

**CONCLUSIONS:**

Cigarette smokers with a short TTFC (≤30 min) had significantly higher levels in VOCs, PAHs, and cadmium, but the association was not mediated by cigarettes smoked per day.

## INTRODUCTION

Approximately 28.3 million smokers are at risk of smoking-related diseases^1,2^. Despite the prevalence of cigarette smoking decreasing to 11.5% in 2021^1-3^, it remains the leading cause of preventable diseases and death in the United States^1^. Previous research indicates that cigarette smokers with a time to first cigarette (TTFC) after waking up of ≤30 min have significantly higher levels of carcinogen biomarkers in polycyclic aromatic hydrocarbons (PAHs:1-hydroxynapthalene, 2-hydroxynapthalene, 3-hydroxyfluorene, and 2-hydroxyfluorene) and the heavy metal (cadmium) compared to those with a TTFC of >30 min^4^. The close association between TTFC and increased biomarkers in PAHs and cadmium could be a crucial step in understanding smoking behavior and tobacco control, complementing the Food and Drug Administration’s (FDA) current strategy of reducing nicotine content in cigarettes^5^ and banning menthol^6^.

In addition to PAHs and cadmium, volatile organic compounds (VOCs) are also included in FDA’s list of Harmful and Potentially Harmful Constituents (HPHCs)^7^. Therefore, the current study had two purposes: 1) to explore the association between TTFC and urine metabolites of VOCs to expand upon previous research^4^ using data from the NHANES 2015–2016 special sample; and 2) to investigate the potential mediation (mediator: cigarettes per day) between TTFC (exposure) and FDA’s list of HPHCs (outcome), including PAHs, cadmium, and VOCs.

## METHODS

The study used data from the National Health and Nutrition Examination Survey (NHANES) 2015–2016 Special Sample. The NHANES program, initiated in the 1960s, has been conducted for several surveys targeting on a wide range of health topics, including smoking and drug use^8^. For eligibility in the current study, participants must be adult smokers who completed the cigarette use survey and reported information on time to first cigarette use (‘How soon after waking do you smoke?’). The final analytic sample comprised 533 exclusive cigarette smokers, with 59.5% smoking their first cigarettes ≤30 min after waking up and 40.5% smoking first cigarettes >30 minutes after waking up^4^.

Smoking characteristics were obtained from the ‘Smoking–Cigarette Use’ and ‘Smoking–Recent Tobacco Use’ questionnaires, including TTFC (‘How soon after waking do you smoke?’) and the average number of cigarettes smoked per day in the last 5 days. TTFC was rated on a 7–point scale: 1 = ≤5 min, 2 = 6–30 min, 3 = >30 min to 1 h, 4 = >1 h to 2 h, 5 = >2 h to 3 h, 6 = >3 h to 4 h, and 7 = >4 h. TTFC was simplified into a binary variable: ≤30 min vs >30 min for ease of comparison and interpretation^4^.

Urine metabolites of VOCs were available in the NHANES 2015–2016 special sample. Supplementary file Table S1 outlines all 17 urine metabolites of VOCs analyzed in the study, including their parent compound and common names. The following covariates were included to control for any potential confounding effects: gender (male vs female), race/ethnicity (Non-Hispanic White vs Non-Hispanic Black vs Hispanics and all others), age, education level (lower than high school vs high school or higher), body mass index (BMI, kg/m^2^), and ratio of family income to poverty^4^.

Multivariable linear regression models were used to determine the covariate adjusted geometric means for urine metabolites of VOCs. Given the lack of normality of urine metabolites, all VOCs biomarkers underwent natural log transformation to better meet regression assumptions. Multivariable adjusted ratios of geometric means of VOCs were estimated by comparing smokers with TTFC ≤30 min to those with TTFC >30 min. The ratios of the geometric means and their 95% confidence intervals (CIs) were obtained by exponentiating the estimates derived from the linear regression models on log-transformed biomarker levels in VOCs. In addition, urine metabolites of VOCs were creatinine-corrected to obtain covariates adjusted geometric means from the regression models consistent with prior studies using urine biomarkers^9-11^.

All hypothesis tests were two-sided and conducted at the significance level of 0.05 in SAS statistical software version 9.4 (SAS Institute Inc, Cary, NC, USA). SAS SURVEY Procedures (PROC SURVEYMEANS and PROC SURVEYREG) were used to perform all statistical analyses with appropriate weights (from the NHANES 2015–2016 special sample), strata, and clustering variables to account for the complex sampling design of NHANES.

### Mediation analysis

PROC CAUSALMED was used to conduct the mediation analysis. The total effect of exposure (TTFC) on the outcome (PAHs, VOCs, and cadmium) was decomposed to direct effect and indirect effect via the potential mediator (CPD) (Supplementary file Figure S1). For mediation analysis, the original 7–point scale of TTFC was kept: 1 = ≤5 min, 2 = 6–30 min, 3 = >30 min to 1 h, 4 = >1 h to 2 h, 5 = >2 h to 3 h, 6 = >3 h to 4 h, and 7 = >4 h. The covariates included gender, race/ethnicity, education level, age at screening, ratio of family income poverty, and BMI.

## RESULTS

Table 1 lists the adjusted geometric means of urine metabolites of VOCs by TTFC status. There were statistically significant differences in 8 out of 17 urine metabolites of VOCs between cigarette smokers with TTFC ≤30 min vs >30 min. Cigarette smokers with short TTFC (≤30 minutes) presented significantly higher concentrations of: xylene (3-MHA and 4-MHA); N, N-dimethylformamide; acrolein (3HPMA); acrylonitrile; 1,3-butadiene (MHBMA3); isoprene; styrene; and crotonaldehyde than cigarette smokers with TTFC >30 min.

**Table 1:** Adjusted geometric means (GMs) of volatile organic compound metabolites (μg/g creatinine) by time to first cigarette (TTFC)

Volatile organic compound metabolites*		*TTFC (≤30 minutes) Mean (95% CI)*	*TTFC (>30 minutes) Mean (95% CI)*	*Ratio of GMs (95% CI)*	*p*
**Parent compound**	**Common name**				
Xylene	2-MHA	110.5 (98.5–124.0)	95.8 (81.5–112.6)	1.2 (1.0–1.4)	0.11
**Xylene**	**3-MHA and 4-MHA**	759.5 (698.2–826.1)	637.8 (569.2–714.9)	1.2 (1.0–1.4)	**0.04**
Acrylamide	AAMA	142.1 (120.7–167.2)	127.8 (110.0–148.6)	1.1 (1.0–1.3)	0.16
**N– N-Dimethylformamide**	**AMCC**	480.9 (443.8–521.1)	409.9 (365.0–460.4)	1.2 (1.0–1.4)	**0.04**
Cyanide	ATCA	170.8 (150.2–194.3)	144.1 (120.1–172.8)	1.2 (1.0–1.4)	0.07
Toluene	BMA	8.1 (6.9–9.4)	7.2 (5.8–8.8)	1.1 (0.9–1.5)	0.34
1-Bromopropane	BPMA	4.1 (3.0–5.6)	4.1 (2.8–6.0)	1.0 (0.6–1.8)	0.99
Acrolein	CEMA	279.8 (246.4–317.7)	250.5 (217.1–288.2)	1.1 (0.9–1.4)	0.31
**Acrolein**	**3HPMA**	1309.6 (1121.9–1528.7)	922.4 (798.8–1065.1)	1.4 (1.1–1.9)	**0.01**
**Acrylonitrile**	**CYMA**	158.1 (134.8–185.3)	122.3 (104.2–143.5)	1.3 (1.0–1.7)	**0.04**
1,3-Butadiene	DHBMA	464.2 (427.4–504.3)	433.2 (408.8–459.2)	1.1 (1.0–1.2)	0.11
**1,3-Butadiene**	**MHBMA3**	32.0 (26.1–39.3)	23.5 (18.8–29.4)	1.4 (1.0–1.8)	**0.04**
**Isoprene**	**4HMBEMA**	43.4 (35.7–52.8)	26.8 (20.8–34.7)	1.6 (1.1–2.4)	**0.03**
Propylene oxide	2HPMA	73.5 (66.3–81.6)	68.8 (61.5–76.8)	1.1 (1.0–1.2)	0.21
**Styrene**	**MA**	289.7 (273.6–306.8)	258.4 (233.5–286.1)	1.1 (1.0–1.2)	**0.02**
Ethylbenzene, styrene	PGA	347.7 (318.0–380.2)	344.3 (300.7–394.2)	1.0 (0.9–1.2)	0.88
**Crotonaldehyde**	**HPMMA**	1430.8 (1263.4–1620.4)	1045.0 (891.5–1225.0)	1.4 (1.1–1.7)	**0.02**

Data Source: NHANES 2015–2016 Special Sample.

* Adjusted for gender, race/ethnicity, education level, age, BMI, ratio of family income to poverty, and cigarettes per day.

Four PAHs (1-hydroxynapthalene, 2-hydroxynapthalene, 3-hydroxyfluorene, and 2-hydroxyfluorene), 8 VOCs (xylene; N, N-dimethylformamide; acrolein; acrylonitrile; 1,3-butadiene; isoprene; styrene; crotonaldehyde) (Table 1) and cadmium, were selected as mediation analysis outcomes due to significant differences observed in carcinogen biomarkers from prior (4 PAHs + cadmium)^4^ and current studies (8 VOCs from Table 1). Table 2 shows the mediation analysis between TTFC and carcinogen biomarkers. Increased TTFC was associated with decreased levels of carcinogen biomarkers in both Model 1 (without adjusting for confounders) and Model 2 (controlled for confounders) for PAHs [total effect (TE) estimate <0), VOCs (TE estimate <0), and cadmium (TE estimate <0) (Table 2). Specifically, as TTFC increased from level 1 (≤5 min) to level 2 (6–30 min) or from level 3 (>30 min) to level 4 (>1 h to 2 h), the levels of outcome decreased in all carcinogen biomarkers (TE estimate <0) (Table 2). As indicated by the natural direct effect (NDE) p-value, after adjusting for CPD (potential mediator), TTFC (exposure) remained significantly associated with PAHs (NDE p<0.01 in all outcomes), VOCs (NDE p<0.01 in all outcomes except styrene; for styrene p≤0.05) and cadmium (NDE p<0.01). Further, the reduction in estimate from total effect (TE) to NDE was minimal. For 1-hydroxynapthalene, the estimate reductions were 18% [(0.27-0.22)/0.27] and 17% [(0.23-0.19)/0.23] in Model 1 and Model 2, respectively. Overall, given the association between TTFC (exposure) and urine biomarker (outcome) remained significant after adjustment of CPD (potential mediator) and relatively small differences in TE and NDE estimates, the data did not provide sufficient evidence regarding the potential roles played by CPD as a mediator for the association between TTFC and carcinogen biomarkers. However, all indirect effects (NIE) are significant (p<0.05).

**Table 2:** Summary of total, direct, and mediated effects from the modern framework of mediation analysis

Outcome*	*Model^§^*	*TE ^a^ estimate*	*p*	*NDE ^b^ estimate*	*p*	*NIE ^c^ estimate*	*p*	*Percentage mediated*
**PAHs**								
1-Hydroxynapthalene	1	-0.27	<0.001	-0.22	<0.001	-0.05	<0.001	19.72
	2	-0.23	<0.001	-0.19	<0.001	-0.04	0.005	16.08
2-Hydroxynapthalene	1	-0.12	<0.001	-0.08	<0.001	-0.04	<0.001	32.04
	2	-0.11	<0.001	-0.07	<0.001	-0.03	<0.001	29.63
3-Hydroxyfluorene	1	-0.20	<0.001	-0.15	<0.001	-0.05	<0.001	24.30
	2	-0.17	<0.001	-0.13	<0.001	-0.05	<0.001	26.65
2-Hydroxyfluorene	1	-0.18	<0.001	-0.13	<0.001	-0.05	<0.001	26.71
	2	-0.16	<0.001	-0.11	<0.001	-0.04	<0.001	25.90
**VOCs**								
Xylene^d^	1	-0.15	<0.001	-0.10	<0.001	-0.05	<0.001	34.07
	2	-0.14	<0.001	-0.10	<0.001	-0.03	<0.001	25.59
N, N-Dimethylformamide	1	-0.14	<0.001	-0.09	<0.001	-0.06	<0.001	40.11
	2	-0.12	<0.001	-0.08	<0.001	-0.04	<0.001	34.56
Acrolein^e^	1	-0.21	<0.001	-0.14	<0.001	-0.07	<0.001	32.09
	2	-0.18	<0.001	-0.13	<0.001	-0.05	<0.001	28.66
Acrylonitrile	1	-0.22	<0.001	-0.17	<0.001	-0.06	<0.001	25.47
	2	-0.19	<0.001	-0.14	<0.001	-0.05	<0.001	27.94
1,3-Butadiene	1	-0.22	<0.001	-0.16	<0.001	-0.06	<0.001	25.77
	2	-0.19	<0.001	-0.15	<0.001	-0.04	0.003	20.44
Isoprene	1	-0.30	<0.001	-0.23	<0.001	-0.06	<0.001	21.42
	2	-0.26	<0.001	-0.22	<0.001	-0.05	0.003	17.42
Styrene	1	-0.09	<0.001	-0.05	0.01	-0.04	<0.001	40.51
	2	-0.07	<0.001	-0.04	0.05	-0.03	<0.001	42.46
Crotonaldehyde	1	-0.22	<0.001	-0.16	<0.001	-0.06	<0.001	28.11
	2	-0.19	<0.001	-0.15	<0.001	-0.05	<0.001	24.22
**Metal**								
Cadmium	1	-0.15	<0.001	-0.11	<0.001	-0.03	<0.01	21.77
	2	-0.10	<0.001	-0.08	<0.001	-0.02	0.01	20.33

Data Source: NHANES 2015-2016 Special Sample. Direct pathway: Exposure (TTFC) → Outcome (PAH, VOC, or Metal). Mediated or indirect pathway: Exposure (TTFC) → Mediator (CPD) → Outcome (PAH, VOC, or Metal). Exposure: TTFC (Time to First Cigarette); 7–point scale: 1: Within 5 minutes; 2: From 6 to 30 minutes; 3: From more than 30 minutes to one hour; 4: From more than 1 hour to 2 hours; 5: From more than 2 hours to 3 hours; 6: From more than 3 hours to 4 hours; 7: More than 4 hours. Mediator: CPD (cigarettes smoked per day).

* Outcome: natural log transformed and creatinine corrected PAHs, VOCs, and cadmium.

§ Model 1: without controlling confounders. Model 2: controlled for confounders. Covariates: gender, race/ethnicity, education level, age at screening, ratio of family income poverty, BMI.

a TE: total effect.

b NDE: natural direct effect.

c NIE: natural indirect effect.

d Xylene common name: 3-MHA and 4-MHA.

e Acrolein common name: 3HPMA.

Despite being smaller in magnitude compared to direct effects, they are not negligible. Further, the ratio of indirect to direct effect also corroborates the importance of mediation. Therefore, we cannot ignore the mediation effect of CPD.

## DISCUSSION

It was found that smokers with ≤30 minutes TTFC had significantly higher concentrations in 8 out of the 17 VOCs examined compared to smokers with TTFC >30 minutes. The aim was to investigate whether the effect of TTFC on biomarker outcomes was mediated through CPD. As presented in Table 2, this pathway was not mediated by CPD, given that the association between exposure and outcome remained significant after adjusting for the potential mediator (CPD). These findings align with a prior study that reported an earlier TTFC had a significant direct effect on increased 4-(methylnitrosamino)-1-(3pyridyl)-1-butanol (NNAL) levels, but the relation was also not mediated by CPD^12^. However, CPD still plays an important role in mediation, as indicated by all indirect effects (NIE) being significant. Although the indirect effects were smaller in magnitude compared to the direct effects, they are not negligible. The different strengths, frequencies, and intensities of each cigarette puff might explain the increased levels of NNAL between the study by Branstetter et al.^12^ and HPHCs biomarkers (PAHs, VOCs, and cadmium) observed in the present study.

In light of the FDA’s tobacco control policy, which centers on reduced nicotine content (RNC) cigarettes^13,14^ and flavor restrictions such as menthol^6,15^, potential limitations arise concerning the acceptability of RNC cigarettes. Users of RNC often report low satisfaction and experience nicotine withdrawal symptoms with these products^16^. Considering the indirect effects of cigarettes per day (CPD) in increasing biomarkers of tobacco exposure, the current study offers another important aspect for the development of tailored smoking intervention programs. Such strategies might aid in lowering biomarkers of harmful carcinogenic chemicals and reducing health risks for all smokers.

### Limitations

This may be the first mediation study between TTFC and FDA’s list of HPHCs including PAHs, VOCs, and cadmium through examination of a nationally representative sample of cigarette smokers. However, several limitations in the study should be noted. Tobacco smoke is the main source of non-occupational exposure to harmful VOCs in the United States^17^, but they are also found in occupational, environmental, and dietary sources. The present study did not adjust for these sources of VOCs, making the current findings subject to confounding. Second, the generalizability of the findings may be limited by the exclusion of non-daily smokers, as NHANES 2015–2016 special sample only captures data on adult smokers who smoke cigarettes every day. Further, the study findings may not be applicable for smokers residing in countries outside of the United States, given the different policies of tobacco control, smoking behavior, availability and access to cigarettes, and risk perceptions of smoking^18-20^. The regression analysis assumptions might be violated due to the distribution of urine biomarkers in NHANES. In addition to log transformation, robust standard errors can provide better and more reliable estimates. Further, given the complex association between exposure and outcome, creating a latent variable (such as a combination of different VOCs or HPHCs) might better capture and depict the mediation more accurately.

## CONCLUSIONS

Cigarette smokers with a short TTFC (≤30 min) had significantly higher levels in VOCs, PAHs, and cadmium, but the association was not mediated by cigarettes smoked per day.

## Supplementary Material



## Data Availability

The data supporting this research are available from the following source: https://wwwn.cdc.gov/nchs/nhanes/continuousnhanes/default.aspx?BeginYear=2015
