# Supplementary material for: Understanding the mediation effects of cigarettes per day on time to first cigarette and carcinogen biomarkers: National Health and Nutrition Examination Survey 2015–2016
Source: Tob Induc Dis. 2024 Jun 10;22:10.18332/tid/187766. doi: 10.18332/tid/187766 (PMC11163879; doi:10.18332/tid/187766)
Supplement: Supplementary file 1 [file TID-22-103-s1.pdf]

Supplementary file

Table S1. VOC metabolites and their parent compounds

Parent Compound	Common Name	Analyte Name
Xylene	2-MHA	2-Methylhippuric acid
Xylene	3-MHA and 4-MHA	3- and 4-Methylhippuric acid
Acrylamide	AAMA	N-Acetyl-S-(2-carbamoylethyl)-L-cysteine
N, N-Dimethylformamide	AMCC	N-Acetyl-S-(N-methylcarbamoyl)-L-cysteine
Cyanide	ATCA	2-Aminothiazoline-4-carboxylic acid
Toluene	BMA	N-Acetyl-S-(benzyl)-L-cysteine
1-Bromopropane	BPMA	N-Acetyl-S-(n-propyl)-L-cysteine
Acrolein	CEMA	N-Acetyl-S-(2-carboxyethyl)-L-cysteine
Acrolein	3HPMA	N-Acetyl-S-(3-hydroxypropyl)-L-cysteine
Acrylonitrile	CYMA	N-Acetyl-S-(2-cyanoethyl)-L-cysteine
1,3-Butadiene	DHBMA	N-Acetyl-S-(3,4-dihydroxybutyl)-L-cysteine
1,3-Butadiene	MHBMA3	N-Acetyl-S-(4-hydroxy-2-butenyl)-L-cysteine
Isoprene	4HMBEMA	N-Acetyl-S- (4-hydroxy-2-methyl-2-butenyl)-L-cysteine
Propylene oxide	2HPMA	N-Acetyl-S-(2-hydroxypropyl)-L-cysteine
Styrene	MA	Mandelic acid
Ethylbenzene, styrene	PGA	Phenylglyoxylic acid
Crotonaldehyde	HPMMA	N-Acetyl-S-(3-hydroxypropyl-1-methyl)-L-cysteine

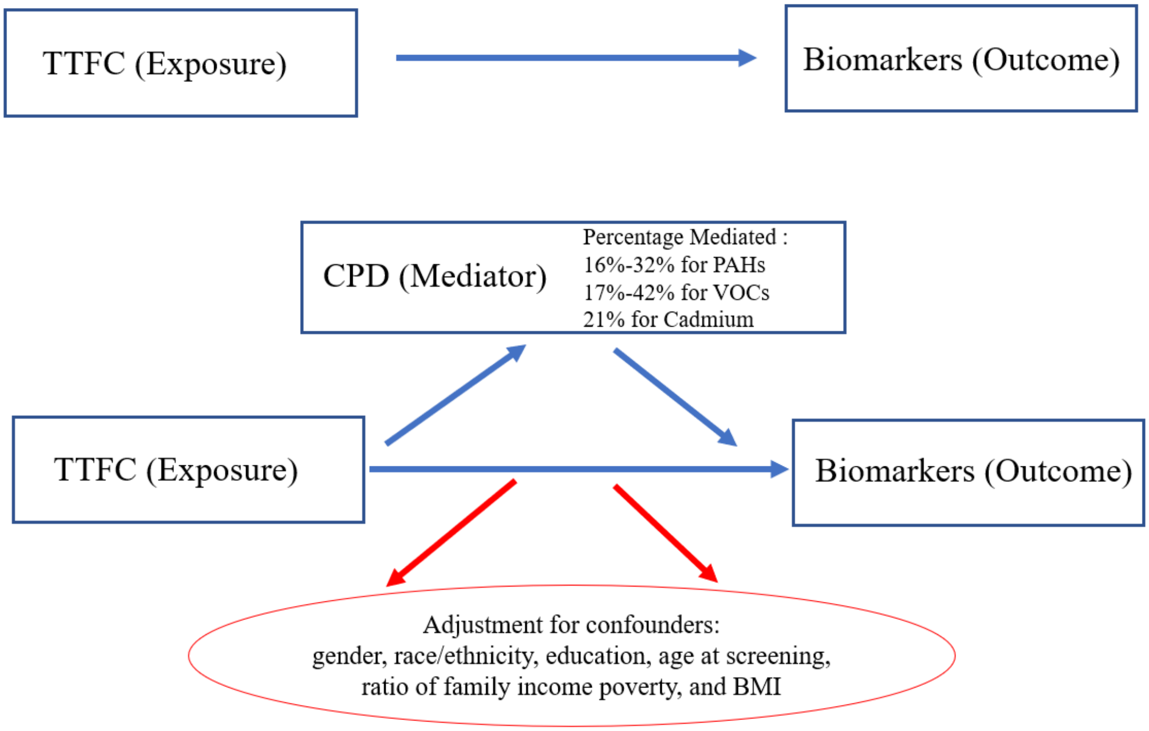

Figure S1. Proposed Mediation of the Association between TTFC and levels of HPHCs biomarkers
Direct pathway: Exposure (TTFC) → Outcome (PAHs, VOCs, or Metal)
Mediated or indirect pathway: Exposure (TTFC)→ Mediator (CPD) → Outcome (PAHs, VOCs, or Metal)
Exposure:
TTFC (Time to First Cigarette) 7-point scale: 1: Within 5 minutes; 2: From 6 to 30 minutes;3: From more than 30 minutes to one hour; 4: From more than 1 hour to 2 hours; 5: From more than 2 hours to 3 hours; 6: From more than 3 hours to 4 hours; 7: More than 4 hours
Mediator: CPD
Outcome: natural log transformed and creatinine corrected PAHs, VOCs, and cadmium
Covariates: gender, race/ethnicity, education, age at screening, ratio of family income poverty, and BMI.

© 2024 Lin W.
